# Capacitive humidity sensing properties of freestanding bendable porous SiO_2_/Si thin films

**DOI:** 10.1038/s41598-022-15955-4

**Published:** 2022-07-08

**Authors:** Soobin Park, Jinmyeong Seo, Jungjoon Park, Inseong Hwang, Han-Seung Lee, Hyunsung Jung, Bongyoung Yoo

**Affiliations:** 1 Department of Materials Science and Chemical Engineering, Hanyang University, Ansan, 15588 Republic of Korea; 2 Department of Architectural Engineering, Hanyang University, Ansan, 15588 Republic of Korea; 3 Electronic Convergence Materials Division, Korea Institute of Ceramic Engineering & Technology, 101 Soho-ro, Jinju, 52851 Republic of Korea

**Keywords:** Sensors and biosensors, Materials for devices

## Abstract

The fabrication of freestanding bendable films without polymer substrates is demonstrated as a capacitive humidity-sensing material. The bendable and porous SiO_2_/Si films are simply prepared by electrochemical-assisted stripping, metal-assisted chemical etching, followed by oxidation procedures. The capacitive humidity-sensing properties of the fabricated porous SiO_2_/Si film are characterized as a function of the relative humidity and frequency. The remarkable sensing performance is demonstrated in the wide RH range from 13.8 to 79.0%. The sensing behavior of the porous SiO_2_/Si film is studied by electrochemical impedance spectroscopy analysis. Additionally, the reliability of the porous SiO_2_/Si sensing material is confirmed by cyclic and long-term sensing tests.

## Introduction

The demands of sensing materials capable of detecting external stimuli and changes are dramatically increasing with the advancement of smart electronic devices in various applications. Especially, bendable, flexible, and stretchable functional sensing materials have been intensively developed by the rapidly growing demand for skin-inspired wearable devices^1–3^. The development of various wearable electronic skin (e-skin) sensors has been required for artificial skins for humans or robots and for individual health monitoring. The human-interactive wearable e-skin devices with skin-like functions that recognize tactile, temperature, and humidity information can be realized with the development of versatile flexible sensors. Artificial tactile systems, equipped with flexible pressure and strain sensors for e-skin devices, have been intensively reported. The research on e-skin devices, including flexible humidity sensors, has focused on their promising role in physiological health monitoring. Most of the reported humidity sensors for e-skin devices have been structures composed of sensing materials on flexible polymer substrates. Polymer substrates, such as polydimethylsiloxane (PDMS), polyethylene terephthalate (PET), and polyimide (PI), have been utilized as flexible supporters, with good mechanical properties, in humidity sensors^4–9^. Huayang et al. developed an e-skin compatible humidity sensor with a two-dimensional WS_2_ film on a flexible PDMS substrate^4^. Shinya et al. fabricated a fast-response and flexible nanocrystal-based humidity sensor on a PI substrate^9^. Additionally, natural polymers, such as silk fabrics, leathers, and papers, have been employed to enhance biocompatibility, elasticity, and flexibility as substrates of e-skin humidity sensors^10–13^. So far, most sensors reported as freestanding flexible humidity sensing materials have been fabricated on flexible substrates or by composite-type sensing materials with flexible materials. Flexible humidity sensors with only freestanding sensing materials, excluding elastic substrates such as polymers, silk fabrics, and papers, have been rarely reported. Xiayu et al. fabricated freestanding dried foam films of graphene oxide for humidity sensing^14^. Qingshen et al. reported a stretchable humidity sensor employing a Ag-PI mixture on PI as a freestanding sensing material^15^. However, the fabrication of flexible and wearable sensors with pure freestanding sensing materials without the aid of flexible polymer substrates is still a challenging issue.

In our previous works, the fabrication of electrochemically exfoliated flexible single crystalline Si films was reported^16,17^. In this paper, based on the previous results for flexible Si films, bendable SiO_2_/Si films for a capacitive humidity sensing device were investigated. The porous SiO_2_/Si film was utilized in a capacitive humidity sensing device. Since hydrophilic porous SiO_2_/layer with high surface area is suitable for the adsorption of water molecules, the SiO_2_ dielectric layer can sensitively detect the humidity levels as a dielectric sensing material for the capacitive sensor. The Si layer was employed as the supporting substrate of the bendable SiO_2_/Si film including the brittle SiO_2_ dielectric layer. The bendable freestanding SiO_2_/Si films, without any flexible substrates, were fabricated by an electrochemical-assisted stripping process of single crystalline Si following by thermal oxidation. Additionally, the surface morphology and thicknesses of the porous SiO_2_ films were controlled by the metal-assisted chemical etching conditions and oxidation time. The capacitive humidity sensing properties of the fabricated freestanding bendable porous SiO_2_/Si films were characterized as a function of relative humidity (RH) and frequency. The sensing behaviors of the porous SiO_2_/Si films were investigated with electrochemical impedance spectroscopy (EIS).

## Results and discussion

**Figure 1:**
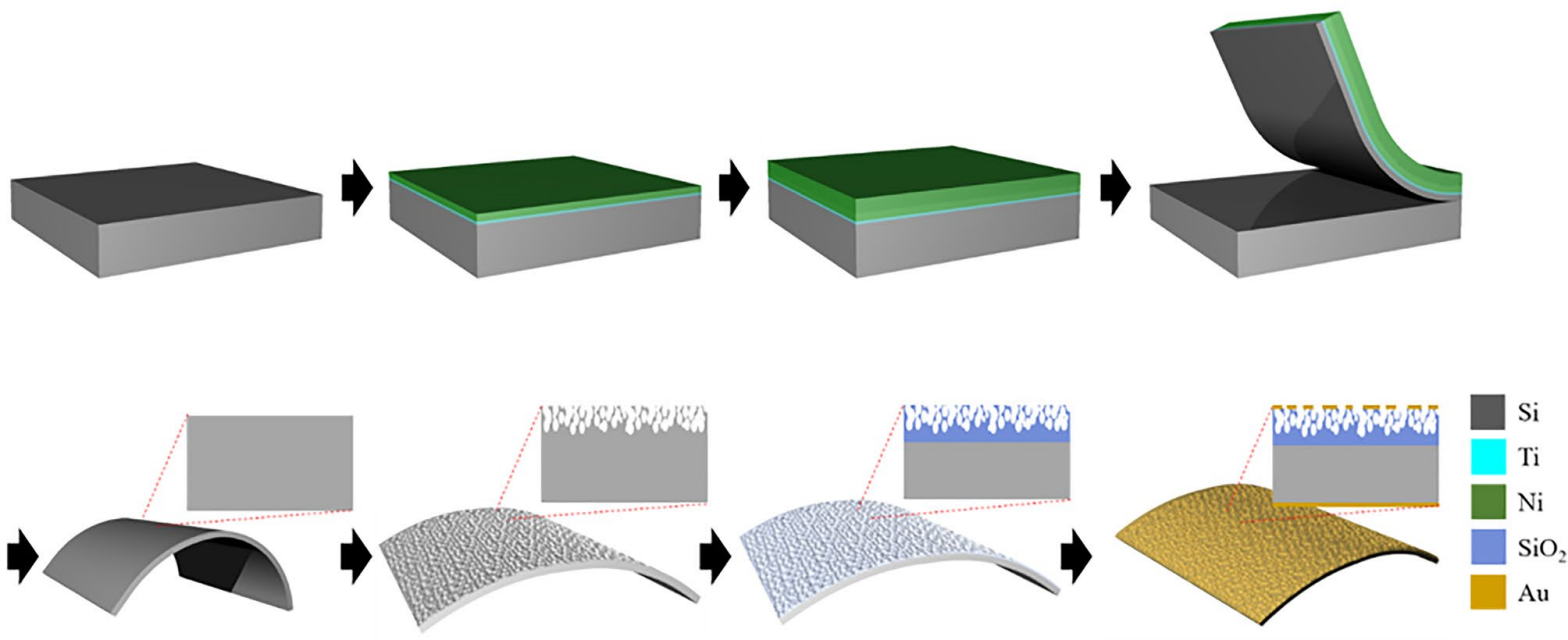
A schematic illustration of the fabrication of a freestanding bendable porous SiO_2_/Si film.

The increasing demand for various flexible electronic devices has encouraged the development of flexible electronic materials. The fabrication of flexible metal oxides, without the aid of polymer substrates, as support structures is still a challenging issue. The fabrication of bendable freestanding SiO_2_/Si films has been demonstrated for a capacitive humidity sensing device. Figure 1 is a schematic illustration of the fabrication procedures for a bendable SiO_2_/Si humidity sensor. First, a Ni/Ti adhesive layer was deposited on a bare Si wafer using an e-beam evaporator. An additional Ni layer was electrodeposited on the e-beam evaporated Ni/Ti adhesive layer. The thin Si layer, with the deposited Ni/Ti layers, was exfoliated by the high tensile stress that was induced in the electrochemical deposition process of the Ni layer. The freestanding flexible single crystalline Si thin film was fabricated by the removal of the deposited Ni/Ti layers on the exfoliated Si layer using chemical etchants. The porous structure of the surface of the exfoliated flexible Si film was formed by a metal-assisted chemical etching process^18^. The surface of the Si film, including the porous Si layer, was oxidized by a thermal oxidation process, resulting in the fabrication of the porous SiO_2_/Si thin film as a freestanding bendable structure. Au/Ti layers on both the top and bottom sides of the SiO_2_/Si film were deposited by an e-beam evaporator as electrodes for a humidity-sensing device.

**Figure 2:**
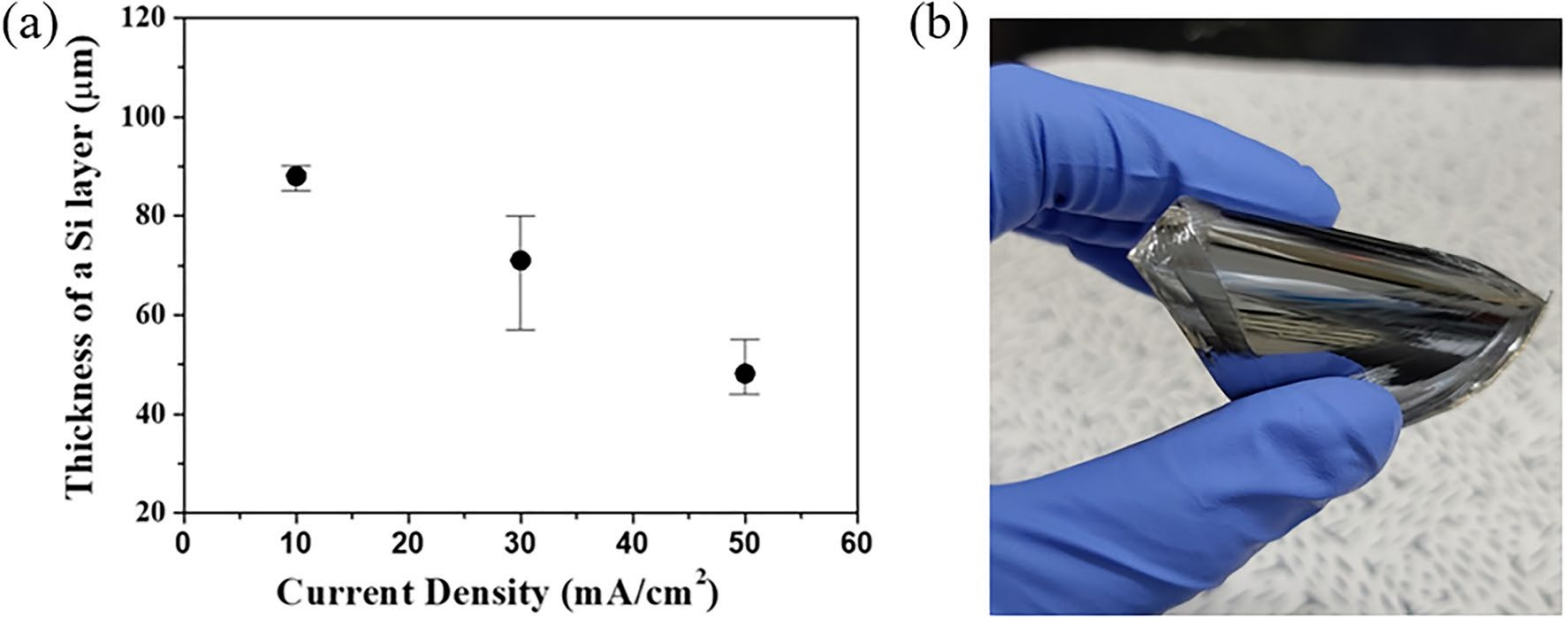
(**a**) The thickness of the exfoliated Si layer as a function of the applied current density in the electrochemical-assisted stripping process, (**b**) the image of exfoliated flexible silicon layer.

The flexible Si thin films prior to the fabrication of the porous SiO_2_/Si thin film were prepared by the exfoliation of the Si wafer due to the tensile stress of the electrodeposited Ni layer. Figure 2a illustrates the thicknesses of the exfoliated Si films as a function of the applied current density to electrodeposit the Ni layers. The thicknesses of the exfoliated layers linearly decreased with the increase in the applied current density for the electrodeposition. The thickness was reduced from approximately 88 μm to 48 μm, as the applied current density was increased from 10 to 50 mAcm^−2^. The previous works have shown that the internal stress of the electrodeposited Ni films increased with the increase in the applied current density^17^. The enhanced film stress was attributed to the increase of the over-potential, the rate of nucleation, and the rate of hydrogen evolution. The enhanced strength of the applied tensile stress decreased the thickness of the spalled Si thin film. The initial spalling time for crack propagation from the edge of the bulk Si wafer might be decreased with an increase in the internal stress intensity of the electrodeposited Ni layer. Therefore, the thickness of the exfoliated Si thin film was reduced by the reduced initial spalling time of the Si thin film. The exfoliated freestanding Si thin film, with well-controlled thickness, can have flexibility, as shown in Fig. 2b.

**Figure 3:**
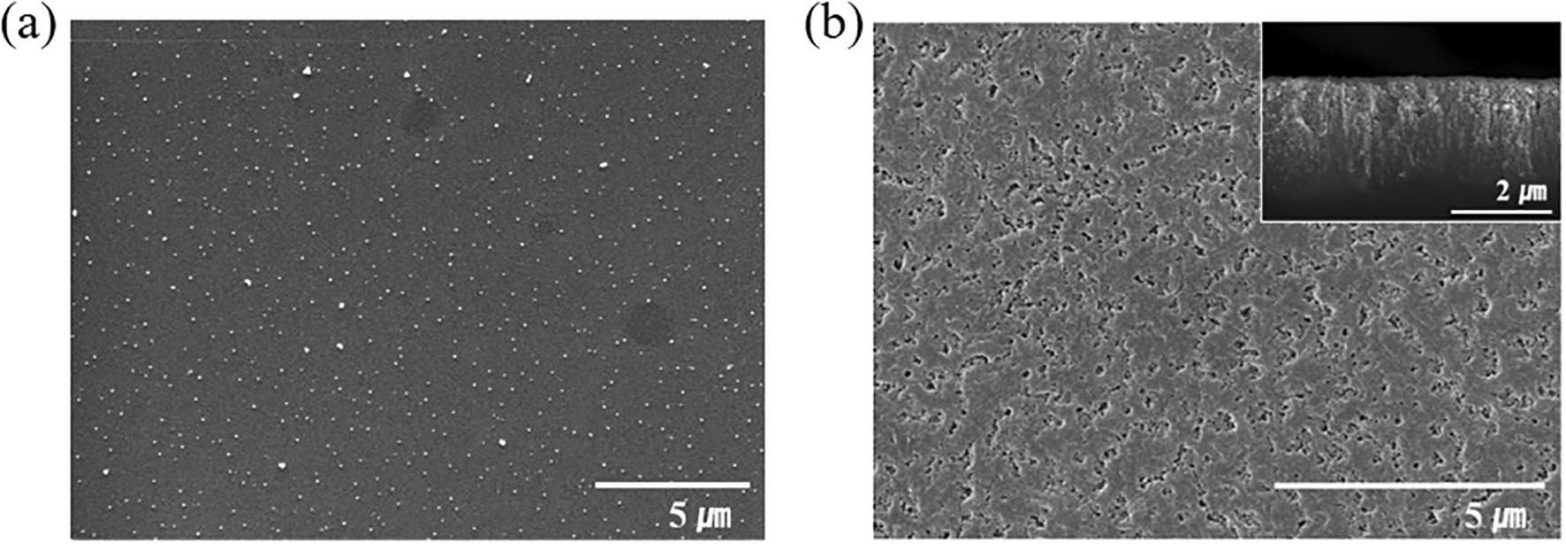
(**a**) SEM image of the electroless deposited Ag nanoparticles on the exfoliated flexible Si film, (**b**) SEM image of the porous Si surface formed by an Ag-assisted chemical etching process (inset: cross-sectional view of the porous Si surface).

The porous structure on the surface of the flexible Si thin film was formed by a metal-assisted chemical etching process^18^. The selective etching of Si was performed at the interface between Si and noble metals. The noble metals act as catalytic cathodes for hole injection in order to accelerate the etching of Si in a HF etchant. Therefore, the morphology of the porous Si structure can be determined by the distribution, size, and shape of noble metals on the Si. Ag nanoparticles were deposited on a Si thin film as the metal catalysts for a Si etching process, as described in Fig. 3a. The good distribution of Ag nanoparticles on the Si thin film was achieved by the controllable nucleation from tin sensitization^19^. Sn^2+^ ions immobilized on the Si thin film can be utilized as nucleation sites for Ag nanoparticles and as reducing agents for the Ag electroless plating. Figure 3b shows the top-view and cross-sectional view (inset) of the porous Si on the Si film prepared by the Ag-assisted chemical etching process. The porous Si layer was etched for 6 h had a thickness of approximately 2.3 μm. The morphologies and the thicknesses of the porous Si layers as a function of the metal-assisted chemical etching times are described in Fig. S1. The thickness of the porous Si layers linearly increased with an increase in the etching time from 30 min to 6 h.

**Figure 4:**
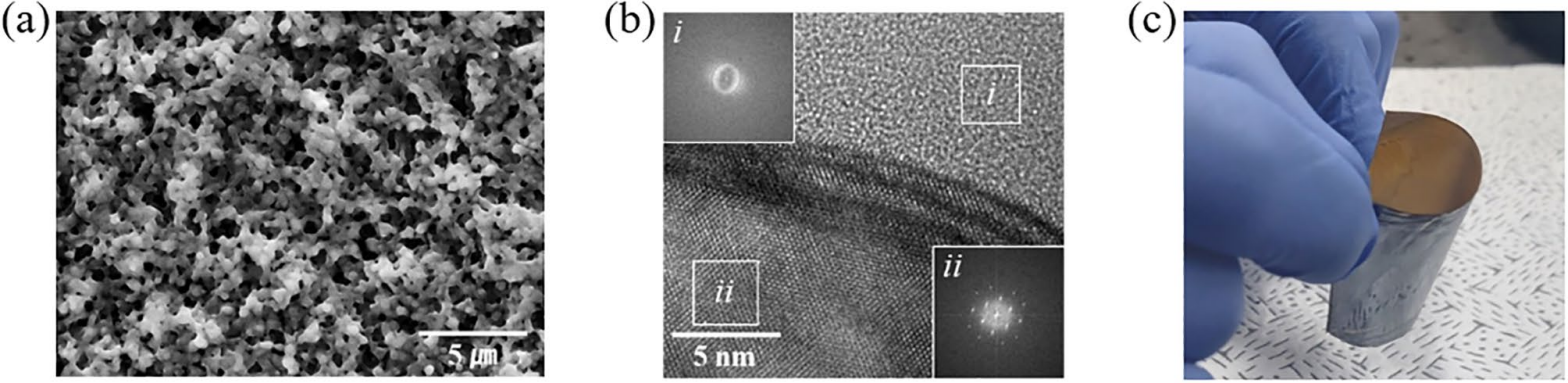
(**a**) SEM image of the porous SiO_2_ surface on a bendable SiO_2_/Si film, (**b**) HRTEM image of the interface of the SiO_2_/Si film (insets: FFT SAED patterns of the SiO_2_ part (*i*) and Si part (*ii*)), and (**c**) the image of the freestanding bendable porous SiO_2_/Si film.

For the fabrication of freestanding bendable SiO_2_/Si films as capacitive humidity sensors, the Si thin film, including the porous Si layer, was annealed at 1000 °C for 6 h in the air gas including 21% O_2_. Figure 4a shows the morphology of the oxidized SiO_2_ on the surface of the bendable SiO_2_/Si film. The oxidized SiO_2_ layer was formed at the surface of the porous Si structures. The thickness of the SiO_2_ layers on the fabricated Si thin film, with a porous surface, can be assumed by the thicknesses of SiO_2_ layers oxidized in bare Si wafers under the same condition at 1000 °C in air. The thicknesses of the SiO_2_ layers formed on Si wafers as a function of annealing time were measured by an ellipsometer, as described in Fig. S2. The SiO_2_ layer formed in the porous Si structure might be expected to be thicker than the thickness of 145.6 nm in the SiO_2_ layer oxidized on a bare Si wafer for 6 h, due to the increased surface area for the oxidation. The cross section of the SiO_2_/Si film was analyzed by high-resolution TEM (HRTEM), as shown in Fig. 4b. The interface of the two layers, consisting of SiO_2_ in the amorphous phase and Si in the single crystalline phase, was clearly distinguished in the HRTEM image. The Si layer prepared by the exfoliation of a single crystal Si (1 0 0) wafer clearly maintained the well-oriented lattices. The HRTEM image of the oxidized SiO_2_ layers unable to observe any structure in the amorphous phase. Additionally, the amorphous SiO_2_ structure and the single crystal Si structure were confirmed with the fast Fourier transform converted from selected area electron diffraction patterns (FFT SAED), as shown in the insets (*i* and *ii*) of Fig. 4b, respectively. The image of a freestanding bendable SiO_2_/Si film including a metal oxide layer is shown in Fig. 4c.

**Figure 5:**
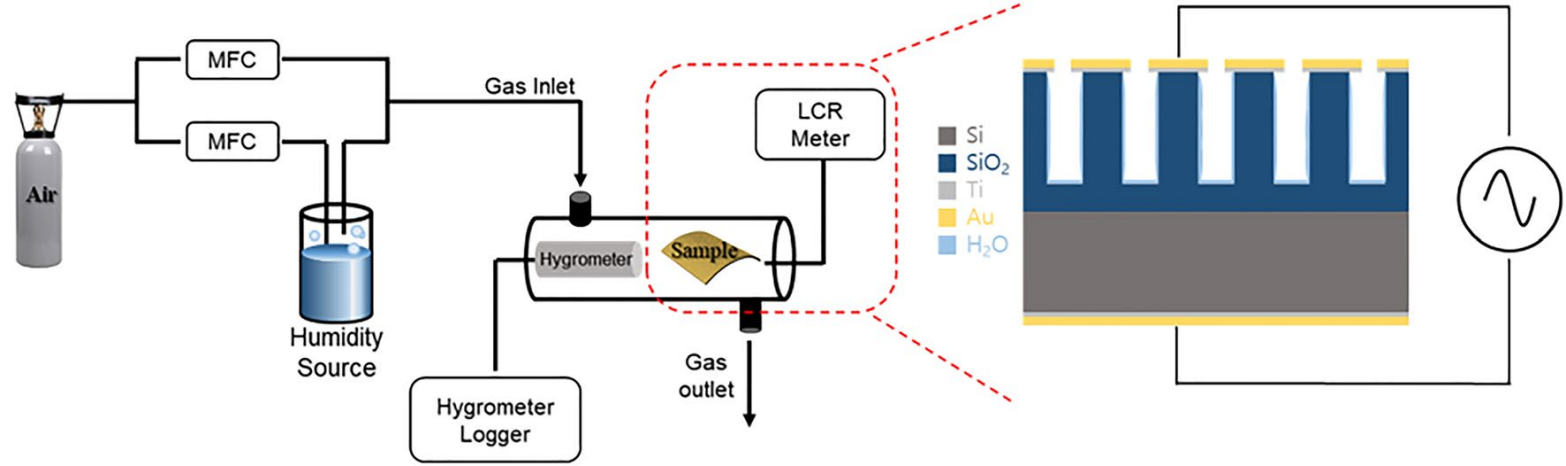
A schematic illustration of the humidity sensing environment.

**Figure 6:**
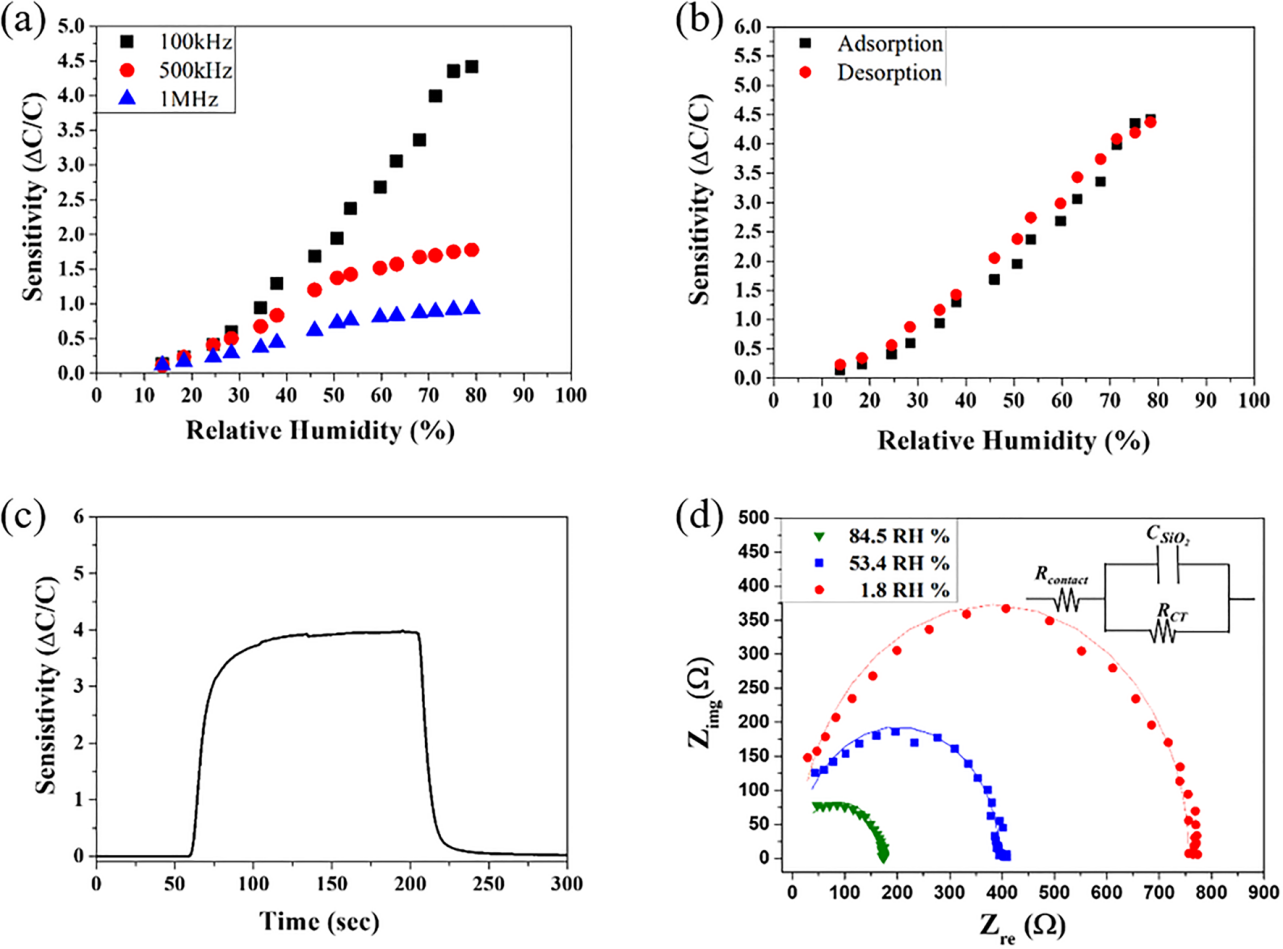
(**a**) Humidity sensing properties as a function of RH at the frequencies of 100 k, 500 k, and 1 MHz, (**b**) hysteresis characteristics for the adsorption and desorption at a frequency of 100 kHz, (**c**) a single sensing curve at 100 kHz in 71.4% RH, and (**d**) Nyquist plots for the RH levels of 1.8%, 53.4% and 84.5% (inset: the equivalent circuit model of the porous SiO_2_/Si film).

The capacitive humidity sensing performance of the fabricated porous SiO_2_/Si film was characterized by controlling the frequency and RH. To measuring the variation of the capacitance of the SiO_2_/Si film, Cu wires were connected with Au electrodes and were deposited on the top and bottom sides of the fabricated SiO_2_/Si film. The variation of the capacitance between the top and bottom of electrodes was measured to characterize the humidity sensing properties. The SiO_2_/Si film was transferred to a humidity sensing chamber. Figure 5 is a schematic illustration of the humidity sensing environment. The humidity of the chamber was controlled by changing the ratio of dry gas to wet gas. The capacitance of the porous SiO_2_/Si sensing material with leak conduction can be described by Eq. (1), where *C*_0_, *ε*_r_, *γ*, *ω*, and *ε*_0_ are the capacitance, and relative dielectric constant of and ideal capacitor, conductance, angular frequency, and permittivity of free space, respectively^20^. The capacitance of the sensing materials is proportional to the reciprocal of the angular frequency, 1/*ω*, and the conductance, *γ*. The sensitivity for the humidity sensing response was calculated by the following Eq. (2), where C_RH_ and C_dry_ were the capacitance under the varied RH and the capacitance of the lowest RH of 1.63%, respectively. Figure 6a shows the sensitivity of the fabricated porous SiO_2_/Si film as a function of frequency and RH. The frequency was varied as 100 kHz, 500 kHz, and 1 MHz, and the RH ranged from 13.8 to 79.0%. As described in Eq. (1), the increase in the applied frequency shows a reduced sensitivity in the range of high RH. No sensing properties were demonstrated for frequencies higher than 1 MHz. The adsorbed water molecules on the sensing material under high frequencies, with a quickly changing electrical field, have insensitive polarization and a reduced dielectric constant^21^. The humidity sensitivity of the porous SiO_2_/Si film linearly increases with an increase in RH. The variation of capacities for the humidity sensing response in the porous SiO_2_/Si film is attributed to the varied conductance from water molecules adsorbed on the surface of porous SiO_2_. Therefore, the enhanced sensitivity in high RH conditions can be explained by the increased polarization of the adsorbed water molecules on the surface of the porous SiO_2_ in the electrical field.

1 $$C=\left({\varepsilon }_{r}-i\frac{\gamma }{\omega {\varepsilon }_{0}}\right){C}_{0},$$

2 $$\mathrm{Sensitivity}=\frac{({C}_{RH}-{C}_{dry})}{{C}_{dry}}.$$

Figure 6b shows the hysteresis characteristics for the capacitive humidity sensing response of the fabricated porous SiO_2_/Si film. The solid square line and solid circle line in Fig. 6b stand for the adsorption of water with increasing RH and for desorption with decreasing RH, respectively. The hysteresis properties were calculated using Eq. (3), where *H*_*e*_, ∆*H*_*max*_, and *F*_*FS*_ are the hysteresis error, a maximum difference of sensitivity between adsorption and desorption at the same RH, and a full-scale output (Sensitivity_at 13.8%_ − Sensitivity_at 78.4%_)^22,23^. The hysteresis error is 5.0% for a RH of 50.1%. The humidity sensitivity after bending test of 5 times was shown in Fig. S3. After the bending test, the sensitivity and linearity were changed to 392% at a RH of 79% and 0.989, respectively.

A single sensing curve at 100 kHz at a RH of 71.4% is shown in Fig. 6c. The response time and recovery time for humidity sensing, defined as the time to reach 90% of the total variation, were 18 s and 30 s, respectively.

3 $${H}_{e}=\pm \frac{{\Delta H}_{max}}{2{F}_{FS}}\times 100.$$

The capacitive humidity sensing performance can be determined by the variation of dielectric constant in sensing materials depending on humidity, as described in Eq. (1). The SiO_2_/Si sensing material with a porous structure has high surface area to contact with water molecules. In dry conditions, the porous SiO_2_ media filled with air have a low dielectric constant. As humidity increased, water vapors were adsorbed on the surface of the porous SiO_2_. The water layers with liquid phase on the surface of SiO_2_ were formed by capillary condensation in the porous structure. The adsorbed water layers in the humid conditions increase the dielectric constant of the porous SiO_2_/Si structure^24–26^. The varied dielectric constants depending on humidity can change total capacitance of the porous SiO_2_/Si sensing material. Therefore, the measured capacitance of the porous SiO_2_/Si humidity sensor can be effectively changed by the variation of the dielectric constant of air to water depending on humid levels.

To prove the humidity sensing mechanism of porous SiO_2_/Si humidity sensor, the humidity sensing behavior of the porous SiO_2_/Si film at various RH levels of 1.8%, 53.4%, and 84.5% was investigated with Nyquist plots utilizing EIS analysis, as shown in Fig. 6d. The frequency varied from 1 kHz to 1 MHz with a testing voltage of 2 V. The semicircles were observed in the complex impedance plots regardless of the controlled RH levels. The unaffected semicircular shapes imply similar sensing behaviors of the porous SiO_2_/Si film on the various RH levels. The semicircles represent the behavior for the intrinsic impedance of the porous SiO_2_/Si film, indicating an equivalent circuit of R (CR), as described in the inset of Fig. 6d. The impedance spectra of the fabricated porous SiO_2_/Si film didn’t show the inclined lines representing a Warburg impedance due to the ion diffusion, even at high RH. Therefore, the sensing behavior of the porous SiO_2_/Si film in the RH range of 1.8% to 84.5% is dominantly determined by the hopping transfer of protons between adjacent hydroxyl groups in the chemisorbed water molecular layer^20,21,23^. With the increase in RH levels, the resistance, *R*_*ct*_, of the porous SiO_2_/Si film decreases, and the capacitance, *C*_SiO2_, gradually increases, displaying the decrease in the radius and curvature of the semicircle. The sensing behavior from the intrinsic impedance of the bulk SiO_2_/Si film at even high RH might be attributed to the highly porous and rough surface structure of SiO_2_. In contrast with the enhanced sensing properties in the porous SiO_2_/Si film, a SiO_2_ layer formed on a bare Si wafer shows no sensing response to the tailored RH, as shown in Fig. S4.

**Figure 7:**
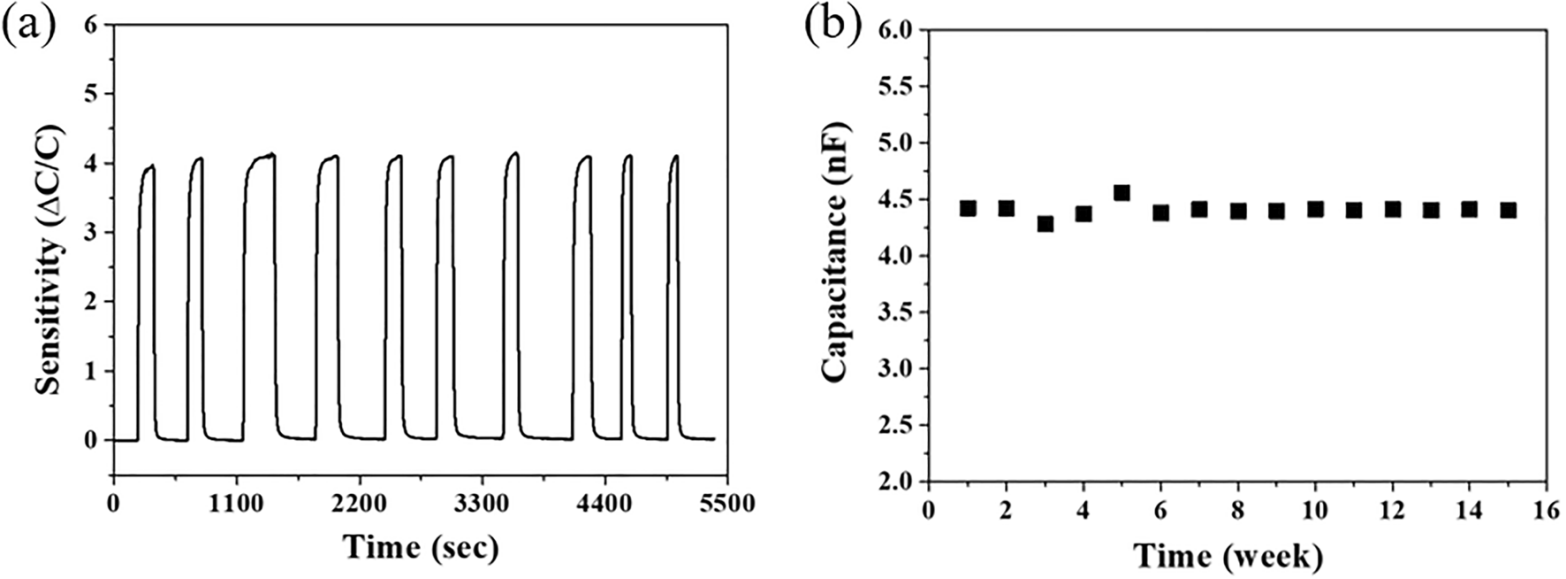
(**a**) The repeated humidity sensing test of 10 cycles at a frequency of 100 kHz for 71.4% RH, and (**b**) long-term stability test of the capacitance as a function of time for humidity sensing at the frequency of 100 kHz for 53.4% RH.

The reliability of the bendable porous SiO_2_/Si film for a capacitive humidity sensing material was investigated, as shown in Fig. 7. The reproducibility for the capacitive humidity sensing properties of the porous SiO_2_/Si films was tested with the sensing of 10 cycles at the frequency of 100 kHz for the RH of 71.4%. The repeated specific sensing signals displayed a reproducible sensitivity with a standard deviation of 0.048. Additionally, the humidity sensing signals at the frequency of 100 kHz for a RH of 53.4% for 15 weeks demonstrate the stable sensing performance for the long-term test. Table S1 in supporting information show a comparison of humidity sensing characteristics of recently reported humidity sensors^7,8,27–35^. The enhanced sensing properties were described in the fabricated porous SiO_2_/Si structure, especially as a flexible capacitive sensor.

## Conclusion

A freestanding bendable SiO_2_/Si film without polymer substrates was fabricated for a capacitive humidity sensing material. The flexible Si film was prepared by a simple electrochemical-assisted stripping process that was a non-vacuum and low-temperature process. The thickness of the exfoliated single crystalline Si film was controlled by the applied current density. The porous structure on the surface of the flexible Si film was formed to enhance the humidity sensing properties by the Ag-assisted chemical etching process. Finally, the freestanding bendable porous SiO_2_/Si film was obtained by the oxidation of the surface of the porous Si film. The remarkable humidity sensing performance of the fabricated porous SiO_2_/Si film was observed with varied frequencies over a wide range from 13.8% RH to 79.0% RH. The capacitance of the porous SiO_2_/Si sensing materials was proportional to the reciprocal of the angular frequency and the RH. The response time and recovery time of humidity sensing for a RH of 71.4% at 100 kHz were 18 s and 30 s, respectively. The hysteresis error between the adsorption and desorption of water molecules indicated 5.0% for a 50.1% RH. The EIS analysis showed that the humidity sensing behavior of the porous SiO_2_/Si film might be dominantly attributed to the hopping transfer of protons in the chemisorbed water molecules. Additionally, the stable sensing behavior in the cyclic and long-term tests was confirmed. Consequently, a highly efficient and stable sensing performance for a wide RH range was demonstrated with the freestanding bendable porous SiO_2_/Si film. The fabricated porous SiO_2_/Si and Si film have attractive properties such as highly textured surfaces enabling lowering of reflectance, the porosity of layer, high surface area, and especially flexibility. The freestanding bendable SiO_2_/Si and Si can be widely utilized in various applications, such as energy storage, solar cell, and biosensors.

## Experimental section

The flexible Si thin film was prepared by an electrochemical-assisted stripping process. The titanium layer of 50 nm thickness and nickel layer of 200 nm thickness on a p-type (1 0 0) silicon wafer as seed layers were deposited by an e-beam evaporator. For the stripping of the Si layer, the Ni layer was galvanostatically electrodeposited with a controlled current density of 10, 30, and 50 mAcm^−2^ in an electrolyte consisting of 1 M NiCl_2_ and 0.1 M Na_3_C_6_H_5_O_7_ in a pH 4.0 HCl solution. The electrochemical bath was maintained at 40 °C and agitated at 200 rpm. The Ni residues on the exfoliated Si film were removed in the Ni etching solution. The porous surface of the prepared flexible Si film was fabricated by the Ag-assisted chemical etching process. Ag nanoparticles as a catalyst for the etching on the flexible Si film were deposited by dipping for 2 min in a 0.02 M SnCl_2_ solution, followed by dipping for 30 min in a 0.02 M AgNO_3_ solution. The porous surface of the Si film was formed by etching for 6 h in a 5 M HF and 4 M H_2_O_2_ solution. Finally, the bendable porous SiO_2_/Si film was obtained by the oxidation for 6 h at 1000 °C in air. Au/Ti electrodes on both sides of the fabricated SiO_2_/Si film were deposited by an e-beam evaporator to measure the humidity sensing properties. To characterize the humidity sensing properties, Cu wires were connected with Au electrodes, deposited on the top and bottom sides of the fabricated SiO_2_/Si film.

The morphologies and microstructures of the materials were characterized by a field emission scanning electron microscope (FE-SEM, MIRA3, Tescan Co.) and transmission electron microscope (TEM, JEM-2100F, JEOL). A LCR meter (E4980A, Keysight), a humidity source (Duran SL. BOT2084), mass flow controller, and a commercial hygrometer were utilized to analyze the sensing properties. Nyquist plots were measured by EIS with a potentiostat (AMETEK, VersaStat3).

## Supplementary Information

Supplementary Information.

## Data Availability

All data generated or analysed during this study are included in this published article (and its Supplementary Information files).
