# Supplementary material for: Capacitive humidity sensing properties of freestanding bendable porous SiO2/Si thin films
Source: Sci Rep. 2022 Jul 8;12:11689. doi: 10.1038/s41598-022-15955-4 (PMC9270390; doi:10.1038/s41598-022-15955-4)
Supplement: Supplementary file 1 — Supplementary Information. [file 41598_2022_15955_MOESM1_ESM.docx]

Supporting Information

**Capacitive Humidity Sensing Properties of Freestanding Bendable Porous SiO_2_/Si Thin Films**

Soobin Park ^1^, Jinmyeong Seo ^1^, Jungjoon Park ^1^, Inseong Hwang^1^, Han-Seung Lee ^2^, Hyunsung Jung ^3*^, and Bongyoung Yoo ^1*^

^1^ Department of Materials Science and chemical Engineering, Hanyang University, Ansan 15588, Republic of Korea

^2^ Department of Architectural Engineering, Hanyang University, Ansan 15588, Republic of Korea

^3^ Electronic Convergence Materials Division, Korea Institute of Ceramic Engineering & Technology, 101 Soho-ro, Jinju, 52851, Republic of Korea

*Co-corresponding authors: hyunsungjung@gmail.com; byyoo@hanyang.ac.kr


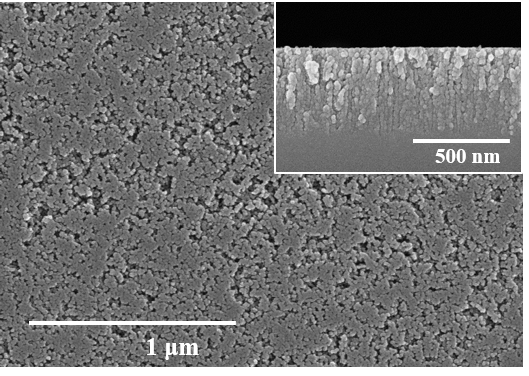

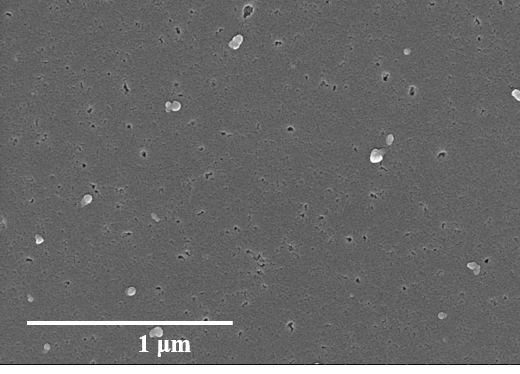
**(**a)

**(b**)


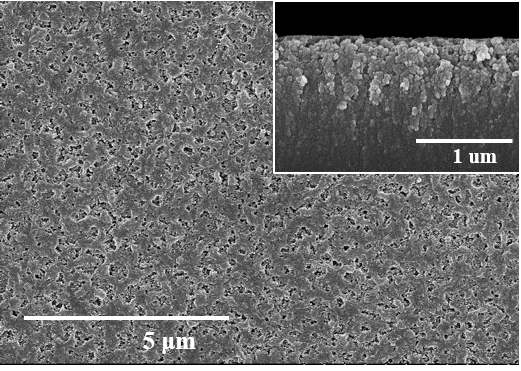

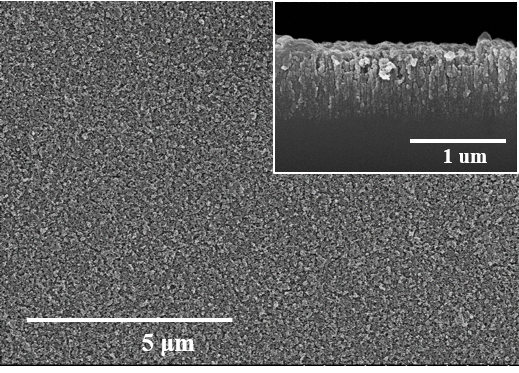
**(c**) **(d**)


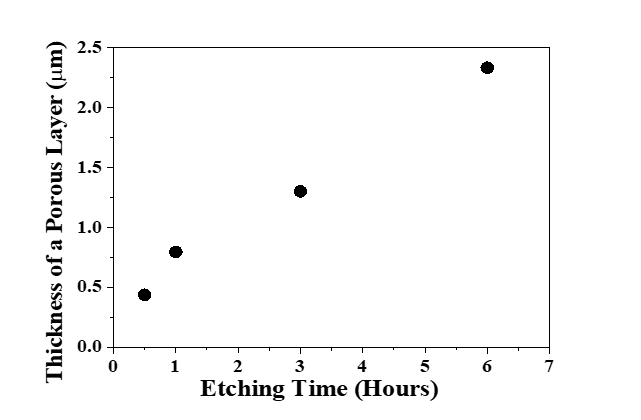

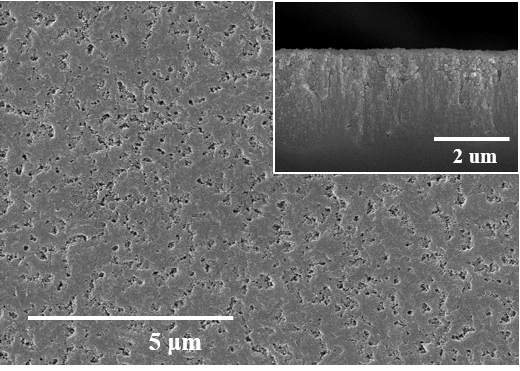
**(**e) **(f**)

Figure S1. The morphologies of porous Si layers depending on the metal-assisted chemical etching times (insets: cross-sectional views of porous Si layers): (a) 10 min, (b) 30 min, (c) 1 hour, (d) 3 hours, (e) 6 hours, and (f) the thickness of porous Si layers as a function of the etching times


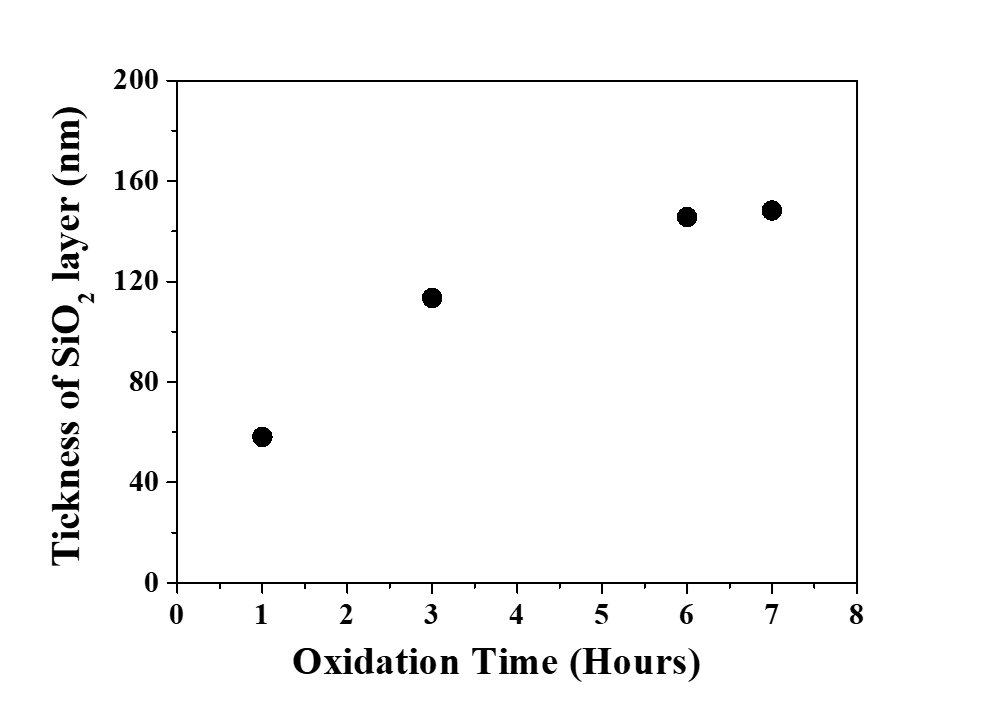


Figure S2. The thickness of SiO_2_ layers formed on Si wafers as a function of oxidation time at 1000 ℃ in the air.


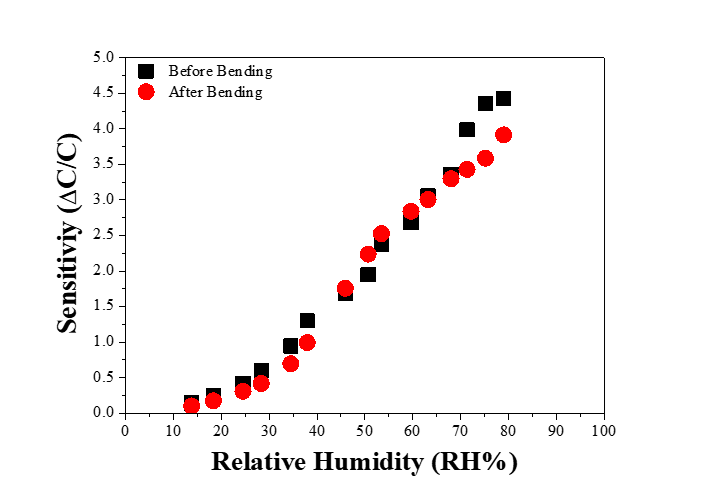


Figure S3. The humidity sensing properties of SiO_2_/Si film before bending test and after bending test of 5 times.


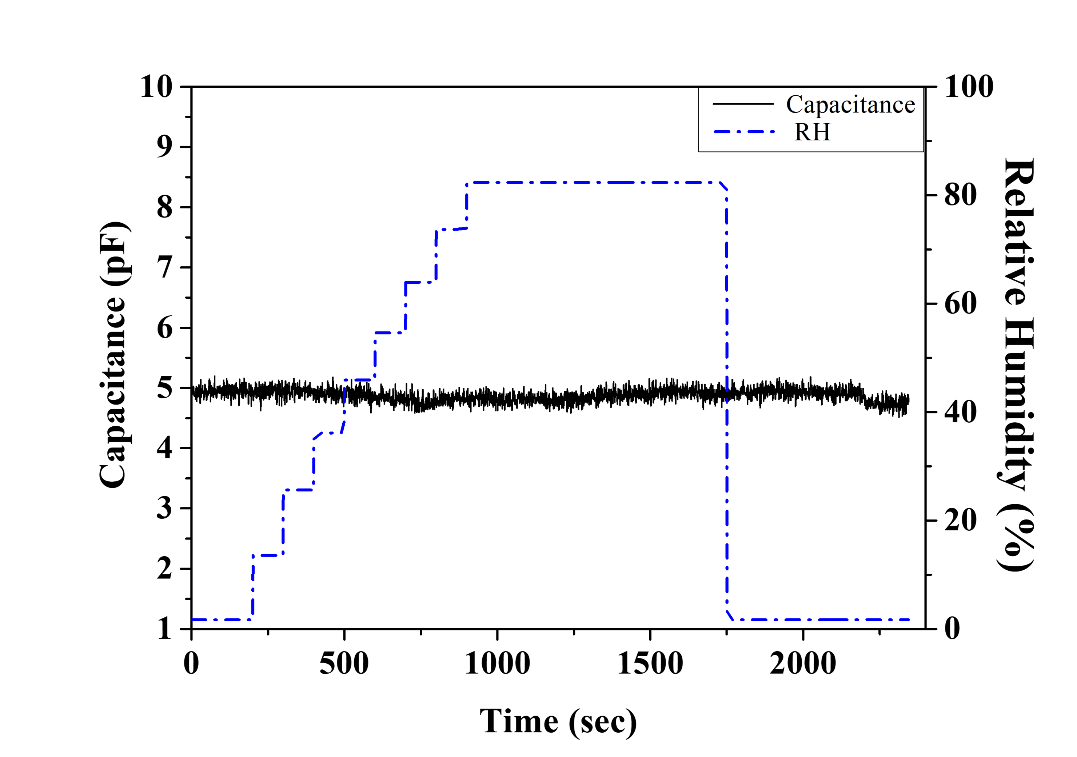


Figure S4. The sensing properties of a SiO_2_ layer formed on a bare Si wafer as a function of the RH.

| \| Sensor Type \| Sensing Material \| Working Range \| Sensitivity* \| Linearity \| Response Time \| Stability \| Flexibility \| Ref. \| \| --- \| --- \| --- \| --- \| --- \| --- \| --- \| --- \| --- \| \| Impedance \| REDOT:PSS \| 0 – 100 \| - \| Non linear \| - \| 20 days \| - \| [27] \| \| Resistive \| ZnO nanosheets \| 11 – 97 \| 645 % \| Non linear \| 42 sec \| - \| - \| [28] \| \| Resistive \| CGO \| 11 – 97 \| 92 % \| - \| 5 sec \| 15 days \| - \| [29] \| \| Resistive \| PET film \| 10 – 90 \| - \| 0.9351 \| 45 sec \| 120 days \| O \| [7] \| \| Resistive \| Carbon Ink \| 2 – 95 \| - \| Non linear \| 56 sec \| 15 days \| O \| [30] \| \| Capacitive \| TiO_2_/polymer \| 10 – 90 \| 32 % \| 0.94 \| 25 sec \| 5 days \| - \| [31] \| \| Capacitive \| ZnO NRs/WS_2_ \| 18 – 85 \| 378 % \| - \| 74.5 sec \| - \| - \| [32] \| \| Capacitive \| P(VDF-TrFFE) nanocone arrays \| 50 – 90 \| - \| 0.985 \| 3.7 sec \| - \| - \| [33] \| \| Capacitive \| Silica-polymer composites \| 11 – 95 \| - \| - \| 165 sec \| 4 weeks \| - \| [34] \| \| Capacitive \| Graphene Oxide \| 30 – 90 \| 209 % \| - \| 38 sec \| 2 weeks \| O \| [35] \| \| Capacitive \| pHEMA \| 35 – 80 \| 172 % \| - \| - \| - \| O \| [8] \| \| **Capacitive** \| **Porous SiO_2_/Si** \| **10 - 80** \| **442 %** \| **0.99** \| **18 sec** \| **15 weeks** \| **O** \| **This work** \|   * for resistive sensor, S=(ΔR/R_0_)*100 and for capacitive sensor, S=(ΔC/C_0_)*100  Table S1. Comparison of humidity sensing characteristics of recently reported humidity sensors. |
| --- | --- | --- | --- | --- | --- | --- | --- | --- | --- | --- | --- | --- | --- | --- | --- | --- | --- | --- | --- | --- | --- | --- | --- | --- | --- | --- | --- | --- | --- | --- | --- | --- | --- | --- | --- | --- | --- | --- | --- | --- | --- | --- | --- | --- | --- | --- | --- | --- | --- | --- | --- | --- | --- | --- | --- | --- | --- | --- | --- | --- | --- | --- | --- | --- | --- | --- | --- | --- | --- | --- | --- | --- | --- | --- | --- | --- | --- | --- | --- | --- | --- | --- | --- | --- | --- | --- | --- | --- | --- | --- | --- | --- | --- | --- | --- | --- | --- | --- | --- | --- | --- | --- | --- | --- | --- | --- | --- | --- | --- | --- | --- | --- | --- | --- | --- | --- | --- |
